# Digital Health Interventions to Enhance Tuberculosis Treatment Adherence: Scoping Review

**DOI:** 10.2196/49741

**Published:** 2023-12-04

**Authors:** Sol Lee, Vasuki Rajaguru, Joon Sang Baek, Jaeyong Shin, Youngmok Park

**Affiliations:** 1 Yonsei University Health System, Yonsei University, Seoul, Republic of Korea; 2 Department of Healthcare Management, Graduate School of Public Health, Yonsei University, Seoul, Republic of Korea; 3 Department of Human Environment & Design, Yonsei University, Seoul, Republic of Korea; 4 Department of Preventive Medicine, College of Medicine, Yonsei University, Seoul, Republic of Korea; 5 Institute for Innovation in Digital Healthcare, Yonsei University, Seoul, Republic of Korea; 6 Division of Pulmonary and Critical Care Medicine, Department of Internal Medicine, Severance Hospital, Yonsei University College of Medicine, Seoul, Republic of Korea

**Keywords:** tuberculosis, patient compliance, digital health, medication adherence, text messaging, mobile apps, application, medication, text, scoping review, disease management, chronic disease, communication, feedback, self-management, PRISMA

## Abstract

**Background:**

Digital health technologies are widely used for disease management, with their computing platforms, software, and sensors being used for health care. These technologies are developed to manage chronic diseases and infectious bacterial diseases, including tuberculosis (TB).

**Objective:**

This study aims to comprehensively review the literature on the use of digital health interventions (DHIs) for enhancing TB treatment adherence and identify major strategies for their adoption.

**Methods:**

We conducted a literature search in the PubMed, Cochrane Library, Ovid Embase, and Scopus databases for relevant studies published between January 2012 and March 2022. Studies that focused on web-based or mobile phone–based interventions, medication adherence, digital health, randomized controlled trials, digital interventions, or mobile health and ubiquitous health technology for TB treatment and related health outcomes were included.

**Results:**

We identified 27 relevant studies and classified them according to the intervention method, a significant difference in treatment success, and health outcomes. The following interventions were emphasized: SMS text messaging interventions (8/27, 30%), medicine reminders (6/27, 22%), and web-based direct observation therapy (9/27, 33%). Digital health technology significantly promoted disease management among individuals and health care professionals. However, only a few studies addressed 2-way communication therapies, such as interactive SMS text messaging and feedback systems.

**Conclusions:**

This scoping review classified studies on DHIs for patients with TB and demonstrated their potential for the self-management of TB. DHIs are still being developed, and evidence on the impact of digital technologies on enhancing TB treatment adherence remains limited. However, it is necessary to encourage patients’ participation in TB treatment and self-management through bidirectional communication. We emphasize the importance of developing a communication system.

## Introduction

Until the COVID-19 pandemic, tuberculosis (TB) was the leading cause of death from a single infectious disease, affecting approximately 10.6 million people in 2021 [1]. TB can be cured with appropriate medications; however, treatment adherence is affected by the complexity, tolerability, and long duration of the available regimens. Since low adherence increases the risk of poor treatment outcomes, several interventions have been attempted to enhance TB medication adherence [2].

Digital health interventions (DHIs) are promising for patient-centered care, as they allow for the remote monitoring of patients and can be used to conveniently remind patients to take their medications. Numerous studies have addressed how to enhance medication adherence during treatment by using mobile technologies, such as SMS text messaging [3], directly observed therapy (DOT) [3-5], video calls, phone call reminders [5,6], and web-based reports [3-7]. Studies have reported satisfaction [6-8], accuracy [6-8], acceptable uptake [5,7,8], improved drug adherence [3-5,7,9], higher rates of treatment success [5,7,8], and user acceptance [7-10] with regard to DHIs in TB management.

This review aims to summarize the existing literature on DHIs for TB treatment adherence, classify DHI techniques, identify the different types of interventions and their effects on treatment effectiveness, and evaluate adherence and health outcomes in TB treatment. This study reports on treatment outcomes, self-care management, follow-up, and the value of mobile-based communication activities that aim to improve TB treatment adherence.

## Methods

We followed Arksey and O’Malley’s [11] 5-stage scoping review framework, the PRISMA (Preferred Reporting Items for Systematic Reviews and Meta-Analyses) statement [12], and the Joanna Briggs Institute protocol [13].

### Identifying the Relevant Studies

We conducted a literature search in the PubMed, Cochrane Library, Ovid Embase, and Scopus databases for relevant studies published between January 2012 and March 2022. A comprehensive search strategy was developed to identify relevant studies, which included but was not confined to the following search string: *(“Tuberculosis” OR “TB” OR “Tuberculosis infection”) AND (“RCT” OR “Randomized controlled trial” OR “Experimental study”) AND (“Behavior therapy” OR “Cognitive behavioral treatment” OR “Digital intervention” OR “Digital therapeutics” OR “App-based” OR “Web-based” OR “mHealth” OR “uHealth”) AND (“treatment adherence” OR “medication adherence” OR “selfcare” OR “Management” OR “Persistence” OR “Compliance”)*. The search terms and strategies are presented in Multimedia Appendix 1.

### Eligibility and Exclusion Criteria

We included articles that met the following criteria: (1) published in peer-reviewed journals, (2) included TB treatment adherence and health outcomes as part of the study design, (3) written in English, (4) had full text available, and (5) published between January 2012 and March 2022. Studies were excluded if they were published before 2011 or did not focus on DHIs for TB. Reviews, case studies, reports, letters, conference proceedings, and abstract-only articles were also excluded.

### Study Selection and Data Synthesis

Duplicates were eliminated from each database and recorded in the first stage. The second stage involved reviewing study titles and abstracts to ensure that articles were research studies that focused on digital health technology as a main intervention tool to improve the treatment adherence of patients with TB. The full texts of the articles were scrutinized in the last stage to verify whether they satisfied the key requirements.

Data were extracted by 1 reviewer (SL), and 2 independent reviewers (VR and YP) charted the data on different characteristics, including authors, publication year, country, study design, target population, number of participants, type of DHI, duration, follow-up, outcome measures, and major findings.

The retrieved data suggested that the core attributes of digital intervention strategies fell under the following three domains, which were based on the DHIs found in the selected articles: sending reminders via SMS text messages, monitoring progress, and tracking follow-ups for the self-management of TB treatment outcomes.

### Quality Assessment and Risk of Bias

Two independent reviewers (SL and YP) evaluated the risk of bias as part of the quality assessment, using the Cochrane Collaboration’s tool for assessing the risk of bias (RoB 2 [Risk of Bias 2]; version: August 9, 2019) [14]. The risk of bias was assessed based on 5 domains, and bias scores were assigned (“low risk,” “some concern,” or “high risk”).

## Results

### Search Results

**Figure 1.**
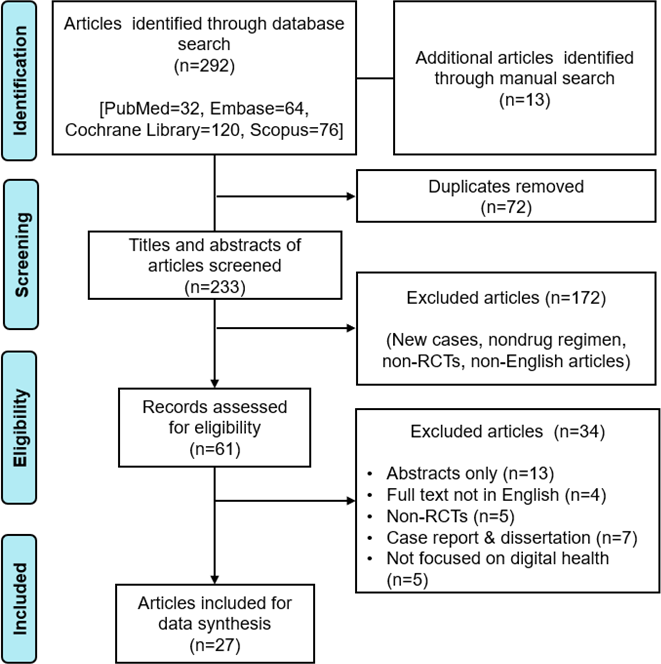
PRISMA (Preferred Reporting Items for Systematic Reviews and Meta-Analyses) flow diagram for selection of articles. RCT: randomized controlled trial.

The literature search retrieved 305 articles; 72 duplicates were excluded, and 172 did not meet the inclusion criteria, based on the title and abstract review. As a result, 61 articles were screened for the full-text review, and 34 were excluded owing to implications regarding the exclusion criteria and unavailability of full texts. Ultimately, 27 studies were finalized for the data synthesis (Figure 1).

### Characteristics of the Selected Articles

Given the novelty of digital health technology in TB treatment, the number of publications was observed to have increased since 2018. A total of 27 articles [15-41] were selected; their characteristics are described in Multimedia Appendix 2. Most of the studies (19/27, 70%) were published in or after 2019 [21,22,24-31,33-41].

With regard to the study designs, 17 studies were randomized controlled trials (RCTs) [15-17,19-21,24-31,34,37,38], 8 were RCT protocols [22,23,32,33,35,36,39,40], and 2 were quasi-experimental studies [18,41]. Further, 13 studies were published in low- and middle-income countries (LMICs), including countries in Africa [18,20,22,23,28,33,35,36,40] and Asia [17,19,25,41]. Digital health technology for TB is actively used in LMICs due to the high prevalence of TB (Multimedia Appendix 3). The combined study population was aged >18 years and included participants who were diagnosed with TB or were taking TB medication. The average number of participants was 400.

### Types of DHIs

**Figure 2.**
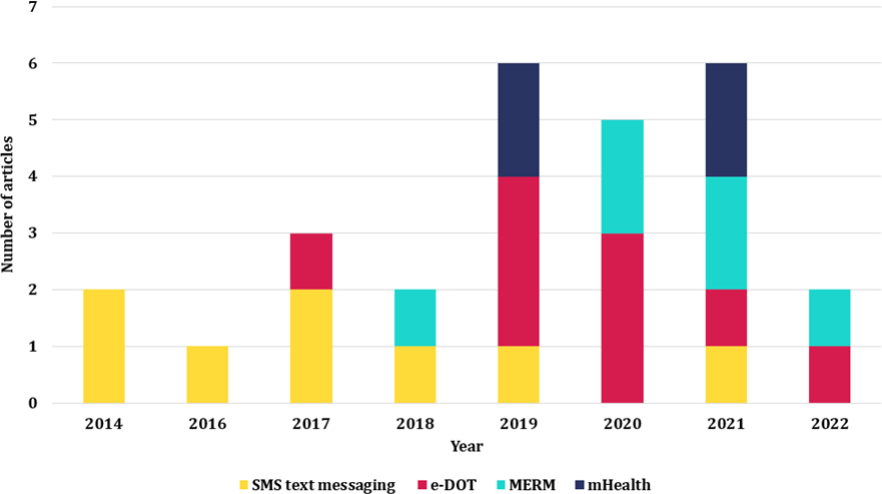
Types of digital health interventions and the number of articles published by year. e-DOT: electronic directly observed therapy; MERM: medication event reminder monitor; mHealth: mobile health.

Table 1 and Figure 2 present the most common technologies used in DHIs, including the duration, frequency, and outcomes of interventions. The commonest DHIs were SMS text messages and reminder messages (8/27, 30%) [15-22], DOT (9/27, 33%) [23-31], medication event reminder monitors (MERMs; 6/27, 22%) [32-37], and mobile apps (4/27, 15%) [38-41]. DOT-based DHIs included video observation therapy (VOT) [25], electronic DOT (e-DOT) [31], and wearable bracelet self-DOT [23]. Some studies evaluated a mix of interventions, including mobile app–based video observations [26,29,30,39], a web intervention [24], WhatsApp (Meta Platforms Inc) [38], and WeChat (Tencent Holdings Ltd) [39]. MERMs [32-37] were also used to determine the feasibility of a web-based follow-up [36] and a mobile-based (ie, evriMED1000 [Wisepill Technologies]) follow-up with phone call [34] reminders to enhance treatment adherence.

**Table 1.** Description of digital health technology tuberculosis (TB) interventions and related outcomes.

Study	Intervention	Main outcome	Secondary outcomes	Duration	Frequency
Bediang et al [15]	SMS text messaging	Treatment success	Treatment adherence, multidrug resistance, and satisfaction	6 mo	Daily
van der Kop et al [16]	SMS text messaging	Treatment success	Treatment adherence and treatment completion	9 mo	Weekly
Mohammed et al [17]	SMS text messaging	Treatment success	Treatment adherence and physical health measures	6 mo	Daily
Hermans et al [18]	SMS text messaging	Risk of LFU^a^ in the first 2 mo of treatment	Treatment success, completion, adherence, satisfaction, and knowledge	2 mo	Other^b^
Farooqi et al [19]	SMS text messaging	Treatment default	TB treatment results according to the WHO^c^^,^^d^	2 mo	Daily
Bediang et al [20]	SMS text messaging	Treatment success	Self-reported adherence regarding attending appointments and satisfaction	6 mo	Daily
Moriarty et al [21]	SMS text messaging	TB treatment results according to the WHO^c^	Smoking cessation, reduction in alcohol use, and treatment adherence	6 mo	Twice weekly
Sahile et al [22]	SMS text messaging	Treatment adherence	ACTG^e^, VAS^f^, and clinic appointment attendance	2 mo	Daily
Huang et al [23]	e-DOT^g^	TB treatment results according to the WHO^c^	Treatment adherence, MGLS^h^, knowledge, and quality of life	6 mo	Daily
Browne et al [24]	e-DOT	Positive detection accuracy	Treatment adherence	Other^i^	Daily
Holzman et al [25]	e-DOT	Treatment adherence	Proportion of all prescribed treatment	Other^i^	Daily
Story et al [26]	e-DOT	Treatment adherence	Treatment outcomes and health-related quality of life	6 mo	Daily
Khachadourian et al [27]	e-DOT	Treatment success	Treatment adherence, depressive symptoms, quality of life, and social support as nonclinical outcomes	4-5 mo	Daily
Crowder et al [28]	e-DOT	Treatment adherence	Reduced risk of LFU and cost-effectiveness	14 mo	Daily
Ravenscroft et al [29]	e-DOT	Treatment adherence	Treatment success at 12 mo	4 mo	Daily
Doltu et al [30]	e-DOT	Treatment adherence	Living conditions, health insurance before TB, previous treatment history, and mode of intensive phase	3 mo	Daily
Burzynski et al [31]	e-DOT	Completed doses and percentage differences between electronic vs in-person DOT^j^	Proportion of medication doses, patient adherence, and quality of care	Other^k^	Daily
Lewis et al [32]	MERM^l^	TB treatment results according to the WHO^c^	Adherence outcomes and cost-effectiveness outcomes	6 mo	Daily
Manyazewal et al [33]	MERM	Treatment adherence and sputum conversion	Adverse treatment outcomes, cost-effectiveness, and usability	15 d	Daily
Ratchakit-Nedsuwan et al [34]	MERM	Treatment success	Treatment adherence and patients’ experiences	6 mo	Daily
Maraba et al [35]	MERM	Treatment adherence	Treatment success, acceptability of the intervention, and cost-effectiveness	18 mo	Daily
Tadesse et al [36]	MERM	Composite unfavorable outcome: treatment failure or death	Longitudinal technology engagement and fidelity to the intervention	6 mo	Daily
Acosta et al [37]	MERM	Treatment success	Treatment adherence, clinical failure, and LFU	4 mo	Daily
NoorHaslinda and Juni [38]	mHealth^m^^,^^n^	Treatment success and treatment adherence	N/A^o^	6 mo	Daily
Wei et al [39]	mHealth^n^	Rate of poor adherence	TB treatment results according to the WHO	6 mo	Daily
Byonanebye et al [40]	mHealth^n^	Treatment success	Treatment success, acceptability of the intervention, and cost-effectiveness	6 mo	Daily
Santra et al [41]	mHealth^n^	Treatment adherence and MGLS	N/A	Other^p^	Daily

a LFU: loss to follow-up.

b Compliance notifications (2, 7, and 11 d after the most recent appointment), appointment notifications (every 2 wk), and educational quizzes (3, 6, 9, and 12 d after the most recent appointment).

c Cured, treatment completed, treatment failed, died, lost to follow-up, not evaluated, or treatment success.

d WHO: World Health Organization.

e ACTG: AIDS Clinical Trial Group adherence questionnaire.

f VAS: visual analog scale.

g e-DOT: electronic directly observed therapy.

h MGLS: Morisky, Green, and Levine Adherence Scale.

i Until TB treatment completion.

j DOT: directly observed therapy.

k Completed 20 medication doses using 1 DOT method, then switched methods for another 20 doses.

l MERM: medication event reminder monitor.

m mHealth: mobile health.

n Smartphone mobile app.

o N/A: not applicable.

p DOT for a minimum period of 30 d and a maximum of 90 d.

### Components of the DHIs and Outcomes

**Figure 3.**
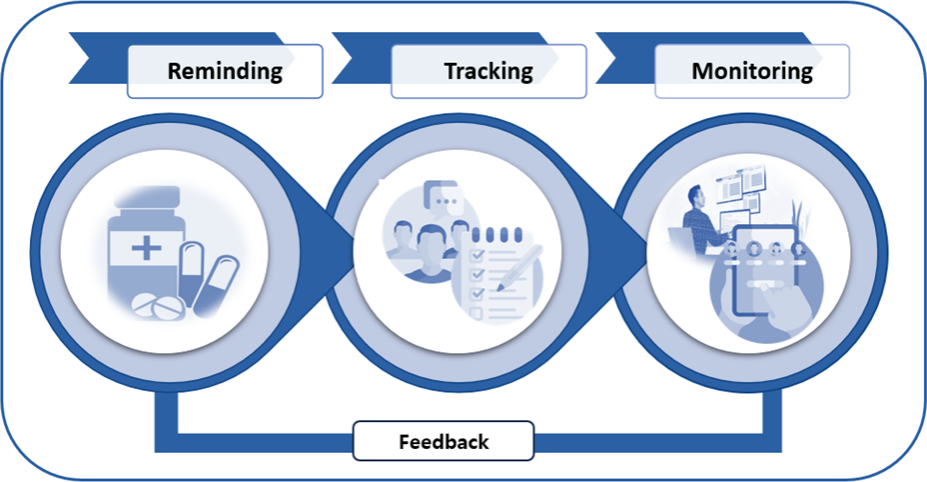
Adherence to tuberculosis treatment is a repeated and ongoing self-management behavior. In this figure, *reminding* refers to reminding patients to take medications as prescribed (ie, correct dose, frequency, and time), *monitoring* refers to using digital health technology (eg, an app) to check whether patients are taking their medication at the prescribed frequency over the initial period, and *tracking* refers to following patients over time to determine whether they taking medications as prescribed [43].

Table 2 presents the components of DHIs that were derived from the primary and secondary outcomes of the selected articles, including (1) sending reminders for treatment adherence via reinforcement SMS text messages [15-22], (2) monitoring treatment adherence by using digital technology [23-31], and (3) tracking treatment adherence through the use of mobile apps and mobile health (mHealth) technology [38-41] via treatment adherence [42] and modified behavior adherence [43] models. Figure 3 presents a modified adherence model.

**Table 2.** Distribution of digital health interventions (DHIs) and related interventions (N=27).

Components and DHIs		Articles, n (%)	References
**Reminding**			
	SMS text messaging	8 (30)	[15-22]
**Monitoring**			
	DOT^a^ (e-DOT^b^, VOT^c^, and WOT^d^)	9 (33)	[23-31]
	MERM^e^	6 (22)	[32-37]
**Tracking**			
	Mobile app and mHealth^f^	4 (15)	[38-41]

a DOT: directly observed therapy.

b e-DOT: electronic directly observed therapy.

c VOT: video observation therapy.

d WOT: wireless observation therapy.

e MERM: medication event reminder monitor.

f mHealth: mobile health.

### Quality Assessment of the Selected Articles

A risk of bias assessment was performed to assess the quality of the selected articles. Only 8 of the 27 articles used an RCT design [17,20,24,26,27,29,31,37]. The risk of bias results are shown in Multimedia Appendix 4 [17,20,24,26,27,29,31,37] and Multimedia Appendix 5.

## Discussion

### Principal Results

This review aimed to identify DHIs related to TB treatment and management. We retrieved the relevant articles from electronic databases by using standard search terms and identified 27 articles published between 2012 and 2022. DHIs for improving treatment adherence were categorized as DHIs for sending reminders [15-22], DHIs for monitoring [23-31], and DHIs for tracking [38-41]. We identified various types of DHIs, including SMS text messaging [15-22], DOT [23-31], MERMs [32-37], and mobile apps [38-41], which improved the effectiveness of self-management, treatment adherence, and the prevention of TB in clinical and community settings.

A total of 19 studies focused on different types of interventions for reminding patients about treatment adherence and included outcomes such as medication adherence [16,21-23,29,33,35,37,41], self-reported survey satisfaction [17,20,22], and appointment attendance [20]. Treatment adherence was primarily accomplished through daily reminder SMS text messages [15-22] and phone calls [22,27,28,41] that requested confirmation of adherence. Furthermore, additional reminders were sent to patients for encouragement or motivation [15,20] if they did not respond within a given time period [16,17,23,28]. Studies also reported sending compliance reminders through daily quizzes [18]; sending reinforcement SMS text messages twice weekly for 12 weeks [21]; and sending system reminders or additional messages to remind patients about the time of medication use [17], confirm daily doses [28], notify patients about a consultation service for their upcoming monthly visits [32], encourage the use of an app [21], and promote self-satisfaction [17,20,22,27]. Rather than demonstrate treatment efficacy, SMS text messaging–based reminder interventions increased patient satisfaction [17,19]. SMS text messaging–based digital technology supports and helps patients and health care professionals to enhance health practices and clinical outcomes. An interactive reminder, such as an SMS text message or video conversation, should be developed according to the required medical monitoring process and incorporated into clinical practice.

Numerous studies have examined the use of DOT to monitor treatment adherence, including 99DOTS [28], VOT [4,29,39], asynchronous VOT [30], wireless observation therapy [24], and e-DOT [40]. DOT also includes treatment regimen monitoring interventions that are based on technology, such as wearable devices [23], mHealth apps [29], and wireless devices [24]. We identified 8 articles that reported e-DOT interventions for TB treatment adherence. Prior studies reported that participants preferred e-DOT over traditional therapy for supporting daily TB medication use during the long-term phase of TB treatment [24,27,29,30]. e-DOT should be tested in areas with a high risk of TB contraction, as e-DOT could greatly enhance the development of programs for treating the disease in LMICs. In addition, VOT interventions for new TB cases were used in combination with a mobile app [26], WeChat (for education and knowledge) [39], and treatment follow-up (with a maximum follow-up interval of 6 months). Story et al [26] reported that VOT resulted in an 80% medication adherence rate in 2 months when compared to DOT, and Ravenscroft et al [29] reported that VOT resulted in about a 45% decrease in nonadherence, which was statistically significant. Further, smartphone-enabled video surveillance of TB therapy has been proven successful and has many advantages over conventional DOT. Wade et al [44] found that VOT increased the proportion of observed treatment doses when compared to DOT; however, the effect on the treatment adherence rate was not statistically significant. Thus, audio- and video-based DHIs may be useful in reducing attrition and improving treatment adherence and health outcomes in acute care settings.

In this review, 4 RCT protocols for MERM-related monitoring interventions were also included [32,33,35,36] to obtain data on the methodological pattern of treatment adherence. Most MERMs are designed to ensure drug compliance, such as evriMED500 [32,33] or evriMED1000 [35,36]. Maraba et al [35] developed an MERM for the daily monitoring of patients and children with drug-susceptible TB during a 6- to 12-month follow-up. Additionally, Ratchakit-Nedsuwan et al [34] conducted a clinical trial of an MERM for patients with pulmonary TB for approximately 6 months; a total of 54 doses were delivered over 70 days, and the adherence rate was approximately 90%. Further, Acosta et al [37] reported that an MERM was significantly more effective than DOT. Hence, we suggest that further RCTs using MERM-based digital intervention strategies should be conducted to enhance TB treatment adherence and clinical outcomes. Since most outcomes were self-reported, additional trials are recommended to determine the accuracy of MERM system–based adherence rates.

Tracking and guiding patients remain important for the follow-up of treatment adherence in a therapeutic context. We found that 4 smartphone-, mHealth-, and mobile app–based digital devices were used to evaluate TB treatment adherence [34,40,41] and acceptability [38]. Patients with pulmonary TB who received intervention through the WhatsApp TB@Clicks module (an mHealth-based DHI) were approximately 4.1 times more likely to have favorable treatment results than a control group [38]. Another DHI for daily drug tracking resulted in drug adherence rates increasing from 85.5% to 96.4% over time [41], and a health-related VOT resulted in decreased nonadherence rates within 4 days [29]. Some apps were combined with a mobile-based pillbox system for a second consultation, resulting in satisfaction and confidence among patients [34]. These outcomes must be incorporated into future clinical trial designs that adopt trustworthy quantitative methods to determine the relative contribution of each digital health technology component.

This review’s findings revealed that DHIs encouraged self-management among patients with TB and empowered them to participate in collaborative discussions during consultations. However, we found that studies on real-time, conversation-based digital technology are lacking; such technology could improve treatment adherence and foster positive health outcomes in various clinical settings. Due to the rapid development of artificial intelligence technologies, including digital tool kits and generative artificial intelligence, 2-way communication–based chatbots in TB treatment may lead to improved self-management in patients with TB.

### Limitations

This review had some limitations. First, our review included studies that focused on treatment outcome–based interventions rather than health care delivery. Therefore, we did not focus on other details, such as TB prevalence, costs, or health insurance. Second, this study focused on the effects of commonly used DHIs on TB treatment outcomes in clinical and community settings. Further studies should determine how DHIs vary between the two contexts and how they interact with multidomain therapies. Third, this study did not specifically describe treatment adherence and self-management. There are no clear differences between the accurate meaning and measurement of treatment adherence in a clinical trial setting and those of self-management in a clinical or community context, and few studies have attempted to provide answers [45-47]. Fourth, many of the included studies (13/27, 48%) were conducted in LMICs because of the high prevalence of TB cases, even though high-income nations have a considerable number of studies. This could be attributed to our study’s selection criteria, such as our criterion for language. Therefore, additional studies are required to identify DHIs across the entire TB care continuum.

### Conclusions

This study examined 27 studies published between 2012 and 2022 and selected the most recent articles. The following three domains were identified from the selected studies: reminding, monitoring, and tracking. The preponderance of treatment adherence was reinforced by mHealth strategies, such as the use of SMS text messaging, mobile apps, mHealth technology, and MERMs. Our findings have implications for TB-related digital health research, which frequently fails to adequately address patients with TB. To preserve treatment adherence and self-care management, patients should have access to real-time, conversation-based interventions (dialogue or communication between patients and health care professionals), such as mobile- or app-based chats, regardless of the restrictions imposed by the COVID-19 pandemic. This scoping review study was conducted before our ongoing chatbot project, which focuses on a mixed methods study on chatbot communication for the treatment adherence of patients with TB. Thus, we emphasize the importance of developing a communication system. DHIs provide several advantages, including improved patient engagement, availability, and accessibility, in addition to lower workloads for practitioners. These results should be considered in the context of national TB control programs and policies to establish a strategy for sustaining TB control and health outcomes. We propose that these developments can significantly improve TB treatment adherence through global collaboration and investment.

## Supplementary material

10.2196/49741 Multimedia Appendix 1 Search strategies.

10.2196/49741 Multimedia Appendix 2 Characteristics of the selected articles.

10.2196/49741 Multimedia Appendix 3 Number of articles published by continent (Africa: Cameroon, Ethiopia, South Africa, and Uganda; Asia: China, India, Malaysia, Pakistan, and Thailand; North America and South America: United States, Peru, and Canada; Europe: United Kingdom and Moldova).

10.2196/49741 Multimedia Appendix 4 Quality assessment and risk of bias based on the five RoB 2 (Risk of Bias 2) domains. Domain 1: randomization process; domain 2: deviations from intended interventions; domain 3: missing outcome data; domain 4: measurement of the outcome; domain 5: selection of the reported result; domain 6: overall.

10.2196/49741 Multimedia Appendix 5 Quality assessment and risk of bias, by intention-to-treat percentage, based on the five RoB 2 (Risk of Bias 2) domains. Domain 1: randomization process; domain 2: deviations from intended interventions; domain 3: missing outcome data; domain 4: measurement of the outcome; domain 5: selection of the reported result; domain 6: Overall.

10.2196/49741 Checklist 1 PRISMA-ScR (Preferred Reporting Items for Systematic Reviews and Meta-Analyses extension for Scoping Reviews) checklist.

## Abbreviations

DHI: digital health intervention
DOT: directly observed therapy
e-DOT: electronic directly observed therapy
LMIC: low- and middle-income country
MERM: medication event reminder monitor
mHealth: mobile health
PRISMA: Preferred Reporting Items for Systematic Reviews and Meta-Analyses
RCT: randomized controlled trial
RoB 2: Risk of Bias 2
TB: tuberculosis
VOT: video observation therapy
