# Supplementary material for: Digital Health Interventions to Enhance Tuberculosis Treatment Adherence: Scoping Review
Source: JMIR Mhealth Uhealth. 2023 Dec 4;11:e49741. doi: 10.2196/49741 (PMC10718480; doi:10.2196/49741)
Supplement: Multimedia Appendix 1 [file mhealth-v11-e49741-s001.docx]

All the search terms filtered by “title, abstract, keywords” and word variations have been searched in each term.

| **Data bases** | **Search terms** |  |
| --- | --- | --- |
| **Pubmed** | | |
| 1 | “Tuberculosis*” OR “TB” OR “tuberculosis infection” OR “active tuberculosis” OR “latent tuberculosis” OR “pulmonary tuberculosis” |  |
| 2 | “digital health” OR “Digital intervention” OR “App-based” OR “Web-based” OR "Mobile app" OR "mobile applications" OR "m-health“OR “mobile message” OR “smart phone” OR “wearable devices” OR “electronic monitoring” OR “eHealth" OR “ voice commander app” |  |
| 3 | DOT* OR directly observed* OR directly observed therapy OR directly observed treatment OR “VOT” OR “VDOT” “videoDOT” OR “eDOT” “video observed treatment,” OR “virtually observed treatment” OR “dose frequency” OR memory aid* OR reminder* OR reinforcement* OR reminder system* OR motivational tool* OR “self-management” OR “self-administered" OR health education*n OR adherence education OR home visit* OR patient education OR counseling OR  “Dose calculator,” |  |
| 4 | anti-tuberculosis treatment* OR anti- treatment* OR Adherence OR compliance OR nonadherence OR non-adherence* OR” concordance” OR “medication adherence” OR “patient adherence” OR “patient compliance” OR Medication Adherence* |  |
| Remarks | [1 AND 2 AND 3] OR [1 AND 2 AND 3 AND 4] |  |
| **EMBASE** | | |
| #1 | ('tuberculosis'/exp OR tuberculosis) AND ('digital health intervention'/exp OR 'digital health intervention') |  |
| #2 | ('tb'/exp OR tb) AND ('mobile app'/exp OR 'mobile app' OR (mobile AND ('app'/exp OR app))) AND ('treatment outcome'/exp OR 'treatment outcome') |  |
| #3 | ('tuberculosis'/exp OR tuberculosis) AND ('digital health intervention'/exp OR 'digital health intervention') AND ('medication compliance'/exp OR 'medication compliance') |  |
| #4 | ('tuberculosis'/exp OR tuberculosis) AND ('medication compliance'/exp OR 'medication compliance') AND ('app based' OR (('app'/exp OR app) AND based)) AND ('therapy'/exp OR therapy) |  |
| #5 | ('tuberculosis'/exp OR tuberculosis) AND ('selfcare'/exp OR selfcare) AND ('drug monitoring'/exp OR 'drug monitoring') |  |
| Remarks | #1 + #2 + #3 + #4 + #5 |  |
| **Cochrane library** | | |
| 1 | “tuberculosis* OR tuberculosis (All fields) OR “pulmonary tuberculosis” |  |
| 2 | “mhealth” OR “mobile phone” OR “smart phone” OR” mobile app” OR “mobile applications” |  |
| 3 | “digital device” OR “digital intervention” OR “wearable devices” OR “tablet” OR “iPhone” OR “monitoring device” |  |
| 4 | “SMS” OR “short message service” OR “MMS” OR “motivation message” |  |
| 5 | “medication adherence” OR “treatment* OR “treatment adherence” OR “treatment follow up” |  |
| Remarks | [1 AND 2 AND 3] OR [1 AND 3 AND 5] OR 1 AND 4 AND 5] |  |
| **Scopus** |  |  |
| 1 | “tuberculosis* OR tuberculosis (All fields) OR “pulmonary tuberculosis” |  |
| 2 | [“mhealth” OR “mobile phone” OR “smart phone” OR” mobile app” OR “mobile applications” ] OR [“digital intervention” OR “wearable devices”] OR [“SMS” OR “short message service” OR “MMS”] OR “ web-based” OR “internet use” |  |
| 3 | “drug therapy” OR medication adherence” OR “treatment outcome* OR “treatment adherence” OR “treatment follow up” OR “self-management” OR “self-care medication” |  |
| Remarks | 1 AND 2 AND 3 |  |
