# Supplementary material for: Digital Health Interventions to Enhance Tuberculosis Treatment Adherence: Scoping Review
Source: JMIR Mhealth Uhealth. 2023 Dec 4;11:e49741. doi: 10.2196/49741 (PMC10718480; doi:10.2196/49741)
Supplement: Multimedia Appendix 2 [file mhealth-v11-e49741-s002.docx]

| Ref | Author (year) /Country | Design | Population (Age/N) | Intervention | | | Control | Outcome | Major finding | |
| --- | --- | --- | --- | --- | --- | --- | --- | --- | --- | --- |
|  |  |  |  | Type | Duration / frequency | Additional reminder |  |  |  |  |
| [15] | Bediang et al.  (2014)  Cameroon | RCT | TPM + patients  Aged ≥18 years  (N=260) | SMS reminders and DOT | 6 months  Daily | Encouraging messages every 2 weeks. | Only DOT | Treatment cure and adherence. | | N/A |
| [16] | van der Kop  (2014)  Canada | RCT | New cases of latent TB [LTBI] patients  Aged ≥19 years  (N=350) | SMS | 9 months  Weekly | Phone call  (If no response within 48hrs) | Standard care. | Self-reported and medication adherence. | | N/A |
| [17] | Mohammed et al.  (2016)  Pakistan | RCT | New cases of TB patients  aged ≥15 years  (N=2207) | SMS | 6 months  Daily | Reminders if no response  First 2 Reminder (within 2 hrs)  3^rd^ or final reminder (>2 hrs)  Phone call (After 7days) | N/A | Treatment success-cured or treatment completion, death, & self-reported adherence | | No significance difference on treatment success  (p = 0·782) and difference in self-report medication differences. |
| [18] | Hermans et al. (2017)  Uganda | Quasi-experimental | TB-HIV patients  Aged ≥18 years (N=582) | SMS | 2 months | Compliance reminder  (2,7&11 days /2weeks)  Educational quizzes  (3, 6, 9 and 12 days) | Standard care | Failure to collect TB treatment for 2 or more weeks and end of TB treatment. | | The SMS intervention was rated as very helpful about 96%. |
| [19] | Farooqi et al. (2017)  Pakistan | RCT | New cases of TB patients  (N=148) | SMS | 2 months  Daily | N/A | Standard care | Treatment default | | No statistically significant difference between intervention and control group (*p* = 0.983) |
| [20] | Bediang et al. (2018)  Cameroon | RCT | Pulmonary TB patients  aged ≥18 years (N=279) | SMS | 6 months  Daily | Weekly motivational text | Standard care | Treatment cure proportion, self-report adherence, attendance of appointments | | 81% Treatment success after 5months.  Very high satisfaction was found in both groups |
| [21] | Moriarty et al. (2019)  South Africa | RCT | Pulmonary TB, current smoker, and alcohol use patients  aged ≥18 years  (N=696) | SMS | 6 months  Twice Weekly | Re-inforcement SMS / twice a week over 12 weeks. | Standard care | Treatment cure, smoking cessation, reduction in alcohol use, and TB medication and anti-retroviral therapy adherence. | | N/A  (Including alcohol use and tobacco smoking) |
| [22] | Sahile et al. (2021)  Ethiopia | RCT  (Protocol) | Four months left of TB therapy patients and aged ≥18 years  (N=186) | SMS and  phone call | 2 months  Daily | N/A | Standard care | Medication adherence, self-report questionnaire | | N/A |
| [23] | Huang et al.  (2017)  China | RCT (Protocol) | TB patients  aged ≥18 years  (N=400) | [BSDOT] | 6 months  Daily | System reminder  (If patient does not send medication taking photo) | DOT | Treatment cured and medication adherence. | | N/A |
| [24] | Browne et al. (2019)  USA | RCT | Isoniazid plus rifampin, with no evidence of drug-resistant TB patients  Aged ≥ 18 years  (N=61) | DHFS, WOT | 12months/  Daily | N/A | DOT  standard care | Binary response of whether the doses were taken or not either wirelessly (WOT) or directly by a health worker (DOT) | | WOT was highly supported for daily medication adherence. It recommended to test high burden TB settings (LMICs) |
| [25] | Holzman et al.  (2019)  India | RCT | TB therapy patients and aged ≥18 years  (N=25) | vDOT (Emocha app) wear | 14weeks /  Daily | N/A | N/A | Treatment adherence, or the proportion of all prescribed treatment | | >70% medication adherence reported after >14 weeks on vDOT follow up |
| [26] | Story A et al. (2019)  UK | RCT | Active TB patients  Aged ≥16 years  (N=226) | VOT Mobile app | 6 months  Daily | N/A | DOT | Successful completion of 80% or more of scheduled treatment observations in the 2 months follow up | | VOT achieved ≥80% in 2 months compared with DOT (31%) and significant (p<0·0001) |
| [27] | Khachadourian et al.  (2020)  USA | RCT | Pulmonary TB patients  Aged ≥18 years  (N=385) | SMS phone calls and DOT | 4-5 month  Daily | N/A | DOT at Outpatient TB centres | Treatment cure, self–reported adherence to TB treatment | | Per–protocol analysis showed that the intervention group successful treatment outcome of 92.0 than DOT |
| [28] | Crowder et al. (2020)  Uganda | RCT | Pulmonary TB patients aged ≥18 years  (N=1890) | DATs, (99DOTS) | 14 months  Daily | Daily dosing confirmation via toll-free phone calls, Weekly check-in via interactive voice response phone calls | N/A | Proportion of patients completing TB treatment | | N/A |
| [29] | Ravenscroft et al.  (2020) Moldova | RCT | TB therapy patients and aged ≥18 years  (N=197) | VOT, mHealth App | 4 months  Daily | N/A | Clinic-based DOT | Adherence to medication | | VOT significantly decreased non-adherence by 4 days (95% ; p<0.01) per 2-week period |
| [30] | Doltu S et al. (2021)  Moldova | RCT | Pulmonary TB patients  aged ≥ 18 years  (N=647) | aVOT | 3 months  Daily | N/A | DOT | Living conditions, Health insurance before TB, Previous treatment history, Mode of intensive phase. | | aVOT group were more likely to have favourable short-term outcome than patients with DOT (p < 0.001). |
| [31] | Burzynski J et al.  (2022)  USA | RCT | Confirmed and Under anti-TB medication patients.  Aged ≥12 years (N=216) | eDOT | eDOT  (20 doses)  Regular (20 doses) | N/A | DOT | Difference between the percentage in-person DOT and with electronic DOT. | | The percentage difference was consistent with both group |
| [32] | Lewis et al. (2018)  China | RCT  (Protocol) | Gene Xpert- Positive and rifampicin-sensitive Pulmonary TB patients  aged ≥18 years (N=1500) | MERM, evriMED 500 | 6 months  Daily | Reminds patients of upcoming monthly visits and to record date and time | Standard care | Treatment cure, adherence, cost-effectiveness. | | N/A |
| [33] | Manyazewal et al.  (2020)  Ethiopia | RCT  (Protocol) | New cases of TB patients  aged 18 to75 years.  (N=144) | MERM, evriMED 500 | 6 months  Daily | N/A | Standard care | Adherence to medication and sputum conversion | | N/A |
| [34] | Ratchakit-Nedsuwan et al.  (2020)  Thailand | RCT | Pulmonary TB patients  aged ≥18 years  (N=100) | Mobile-equipped pill box  (CARE-call system) | 6 months  Daily | Second reminder consultancy service and emergency call service via pill box | Standard care | Medication adherence, patient’s experiences, opinions, and problems | | At a 90% adherence level,  satisfied and confident of the monitoring process |
| [35] | Maraba et al. (2021)  South Africa | RCT  (Protocol) | Drug susceptible TB patients and children  Aged 2–17 and ≥18 years.  (N=2610) | MERM, (evriMED 1000) | 6 months - 12 months  Daily | N/A | Standard care | Medication adherence | | N/A |
| [36] | Tadesse et al. (2021)  Ethiopia | RCT  (Protocol) | Aged 18 years or over, not requiring hospitalization, and providing consent.  (N=4000) | evriMED 1000 and ASCENT web-based app | 6 months  Daily | N/A | Standard care | composite unfavourable outcome measured over 12 months from TB treatment | | N/A |
| [37] | Acosta J et al. (2022)  Peru | RCT | Pulmonary TB patients  aged ≥18 years (N=102) | MERM | 54 doses (70 days)  Daily | N/A | DOT | Treatment cure, Medication adherence | | Treatment success was significantly more frequent in the MERM group than DOT group |
| [38] | Noor Haslinda et al. (2019)  Malaysia | RCT | Newly diagnosed or positive TB patients  Aged 18 to 65 years.  (N=110) | Mobile app (WhatsApp, TB@Clicks) | 6 months  Daily | N/A | TB information via WhatsApp only | Treatment adherence rate to TB treatment and success rate. | | Intervention was 4.1 times as likely to had successful treatment outcome than control group |
| [39] | Wei et al. (2019)  China | RCT  (Protocol) | New cases of TB patients  aged ≥15 years (N=300) | VOT, WeChat | 6 months  Daily | Audio/video-based health education messages by WeChat | Standard care | Rate of poor adherence, measured monthly during treatment as a binary indicator | | N/A |
| [40] | Byonanebye et al.  (2021)  Uganda | RCT  (Protocol) | Xpert Positive and Pulmonary TB patients aged ≥18 years  (N=274) | Mobile CFL-TB  MoTeCH | 6 months  Daily | Calls to registered care providers (buddies) of patients to facilitate DOT | Standard care | Treatment cure, sputum test in the last month of treatment and on at least one previous occasion. | | N/A |
| [41] | Santra S et al. (2021)  India | Quasi-experimental study | TB patients with DOT (over 30days)  aged ≥18 years (N=220) | SMS  Phone calls | 3 months  Daily | a weekly real-time two-way phone call that lasted for 10 min. | DOT | Treatment adherence  4-item (MGLS). | | Medication adherence (daily DOTS regimen) was increased (85.5% ) than DOT |

TB= Tuberculosis; SMS=Short Message service; DOT= Directly Observed Treatment; VOT= Video-observed therapy; eDOT=Electronic directly observed therapy; aVOT =Asynchronous video observed therapy; DHFS= Digital health feedback system; MERM= Medication event reminder monitor; MGLS= Morisky-Green-Levene adherence scale; CFL-TB= Call for Life-TB; MoTeCH =[NoorHaslinda, 2019 #47] technology for community health; LMICs= Low- and middle-income country; USA= United states of America; N/A= Not available
