# Supplementary figures and images for: Digital Health Interventions to Enhance Tuberculosis Treatment Adherence: Scoping Review
Source: JMIR Mhealth Uhealth. 2023 Dec 4;11:e49741. doi: 10.2196/49741 (PMC10718480; doi:10.2196/49741)

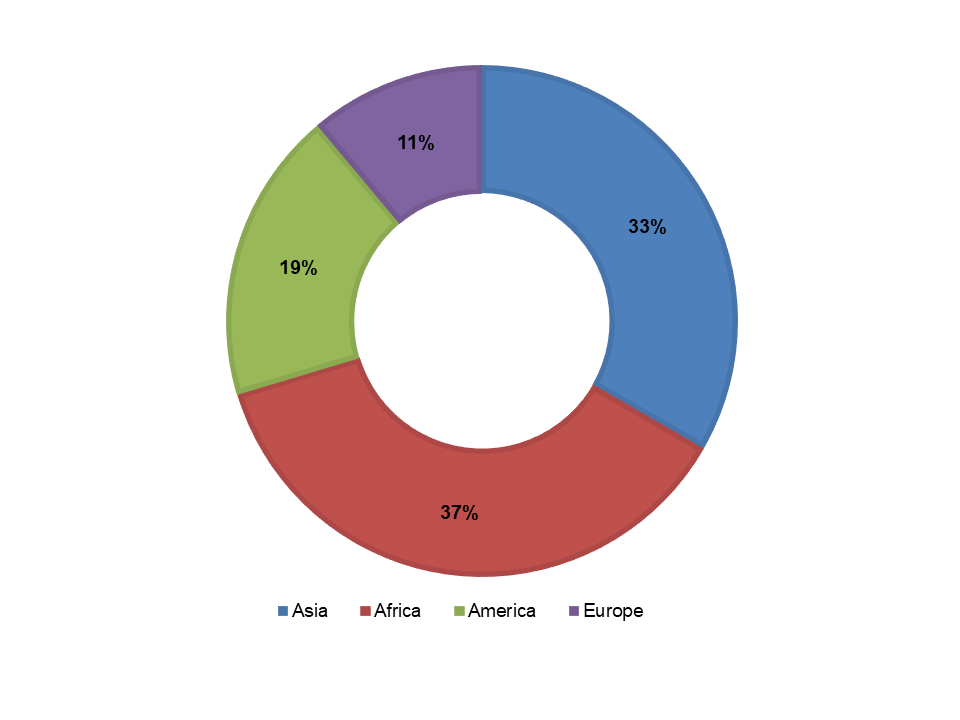

Supplement: Multimedia Appendix 3 [file mhealth-v11-e49741-s003.png]

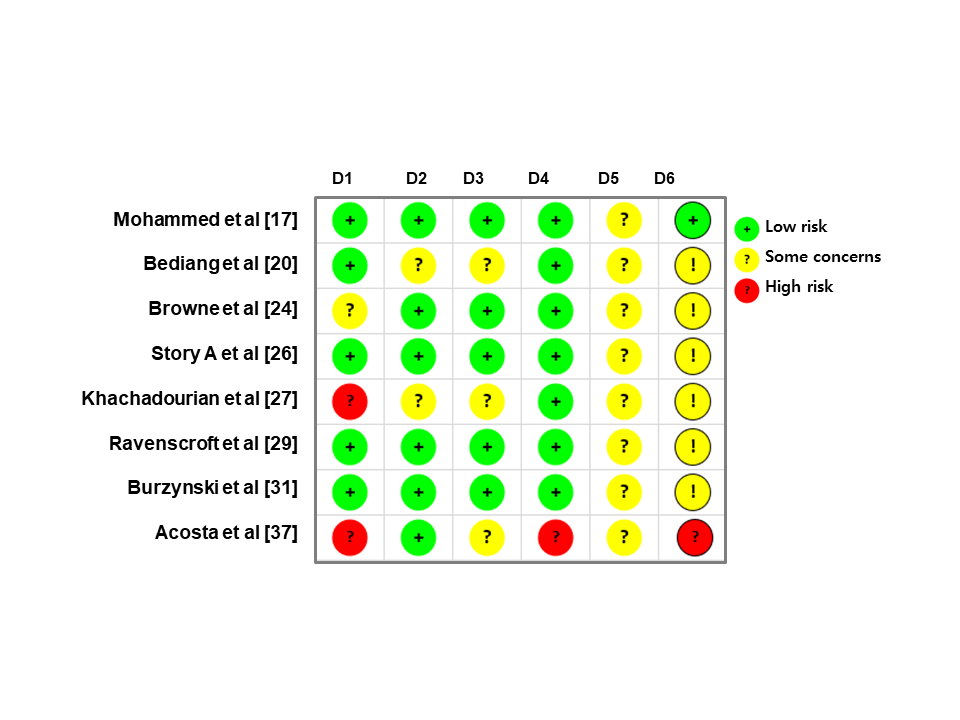

Supplement: Multimedia Appendix 4 [file mhealth-v11-e49741-s004.png]

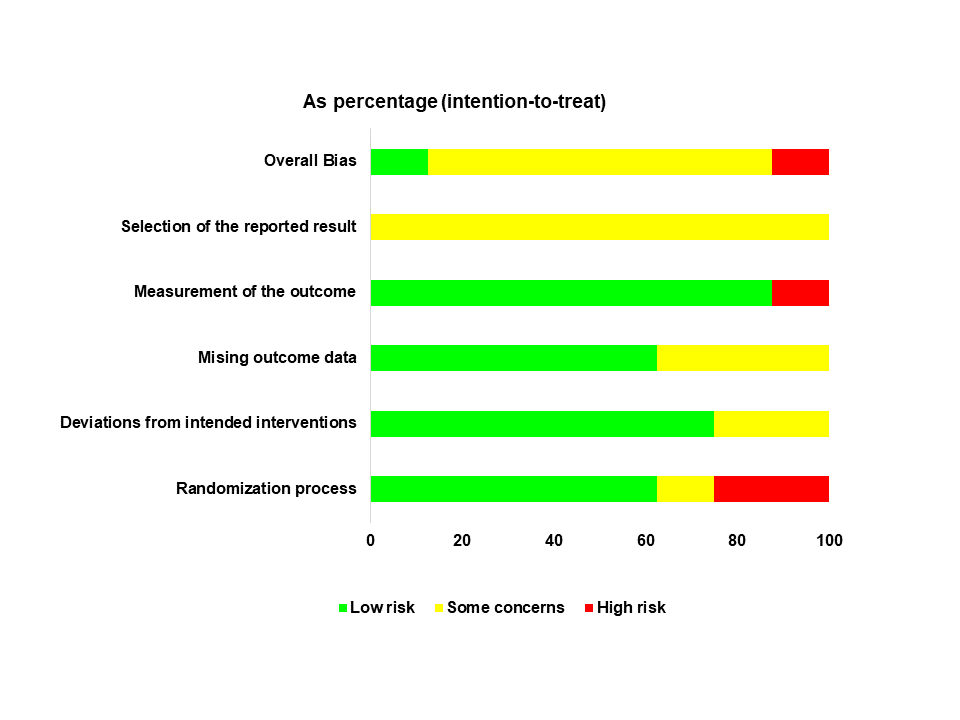

Supplement: Multimedia Appendix 5 [file mhealth-v11-e49741-s005.png]
